# Trisomy 19 ependymoma, a newly recognized genetico-histological association, including clear cell ependymoma

**DOI:** 10.1186/1476-4598-6-47

**Published:** 2007-07-12

**Authors:** Emmanuel Rousseau, Thomas Palm, Francesco Scaravilli, Marie-Magdeleine Ruchoux, Dominique Figarella-Branger, Isabelle Salmon, David Ellison, Catherine Lacroix, Françoise Chapon, Jacqueline Mikol, Miikka Vikkula, Catherine Godfraind

**Affiliations:** 1 Laboratory of Pathology, Université catholique de Louvain, Bruxelles, Belgium; 2 Laboratory of Human Molecular Genetics, de Duve Institute, Université catholique de Louvain, Bruxelles, Belgium; 3 Institute of Neurology, Queen Square, London, UK; 4 INSERM U689 Paris and CEA, Fontenay-aux-Roses, France; 5 Department of Pathology and Neuropathology, La Timone's Hospital, AP-HM, and EA3281 Université de la Mediterrannée, Marseille, France; 6 Laboratory of Pathology, Erasme Hospital, Université Libre de Bruxelles, Bruxelles, Belgium; 7 Northern Institute for Cancer Research, University of Newcastle, Newcastle-upon-Tyne, UK; 8 Laboratory of Pathology, Hôpital Kremelin-Bicêtre, Paris, France; 9 Laboratory of Pathology, CHU-Caen, France; 10 Department of Pathology, Hopital Lariboisière, Paris, France

## Abstract

Ependymal tumors constitute a clinicopathologically heterogeneous group of brain tumors. They vary in regard to their age at first symptom, localization, morphology and prognosis. Genetic data also suggests heterogeneity. We define a newly recognized subset of ependymal tumors, the *trisomy 19 ependymoma*. Histologically, they are compact lesions characterized by a rich branched capillary network amongst which tumoral cells are regularly distributed. When containing clear cells they are called clear cell ependymoma. Most trisomy 19 ependymomas are supratentorial WHO grade III tumors of the young. Genetically, they are associated with trisomy 19, and frequently with a deletion of 13q21.31-31.2, three copies of 11q13.3-13.4, and/or deletions on chromosome 9. These altered chromosomal regions are indicative of genes and pathways involved in trisomy 19 ependymoma tumorigenesis. Recognition of this genetico-histological entity allows better understanding and dissection of ependymal tumors.

## Background

Ependymal tumors include a broad histological and clinical spectrum of lesions presumably derived from ependymal cells contributing to the lining of the cerebral ventricles and the remnants of the central canal of the spinal cord [1]. Their overall incidence is 0.23 cases per 100,000 individuals per year in the United States, with a mean age at diagnosis of 35 years and an overall 5-year survival of 66% [2]. Ependymal tumors represent the seventh most frequent primary brain tumor in adult and the third in children. The last WHO classification comprises WHO grade I myxopapillary ependymoma and subependymoma, WHO grade II ependymoma, and WHO grade III anaplastic ependymoma [1].

In contrast to astrocytic and oligodendroglial tumors, in which molecular alterations associated with tumorigenesis are relatively well established, less is known about molecular changes in ependymal tumors. Monosomy of chromosome 22 and gain of chromosome 7 occur more frequently in spinal cord than in intracranial tumors [3]. In contrast, gain of chromosome 1q, and losses of chromosomes 6q, 9, and 13, are more frequently observed in the latter [3-5]. The involvement of chromosome 9 in ependymal tumors led to study the three 9p21 located tumor suppressor genes, *CDKN2A*, *CDKN2B *and *p14ARF*. Deletions of *CDKN2A *were found in 25% of the investigated tumors [6]. Promoter methylation of *CDKN2A*, *CDKN2B *and *p14ARF *was detected in 20–30% of tumors, with variations according to clinico-pathological characteristics [7].

Here we have analyzed ependymomas which have lost at least 9p and discovered that most of these tumors share architectural features, reminiscent of clear cell ependymoma. We performed array-CGH on a series of such tumors and observed trisomy 19 to be present in all of them. This copy number change was associated with alterations in chromosomes 9, 11 and/or 13. This data allowed us to define a new genetico-histological ependymal tumor entity, the *trisomy 19 ependymoma*. Histologically, they are compact with a clear tumor-to-parenchyma interface, presenting a branched capillary network and regularly dispersed tumoral cells. Clinically, most are supratentorial WHO grade III tumors of the young. This data underscores the heterogeneity within ependymal tumors and the need for further genetic dissection, a mandatory step to establish personalized oncological practice.

## Results

### Chromosome 9 microsatellite analysis of the formalin-fixed and paraffin-embedded ependymal tumor series and association with clinico-pathological parameters

**Figure 1:**
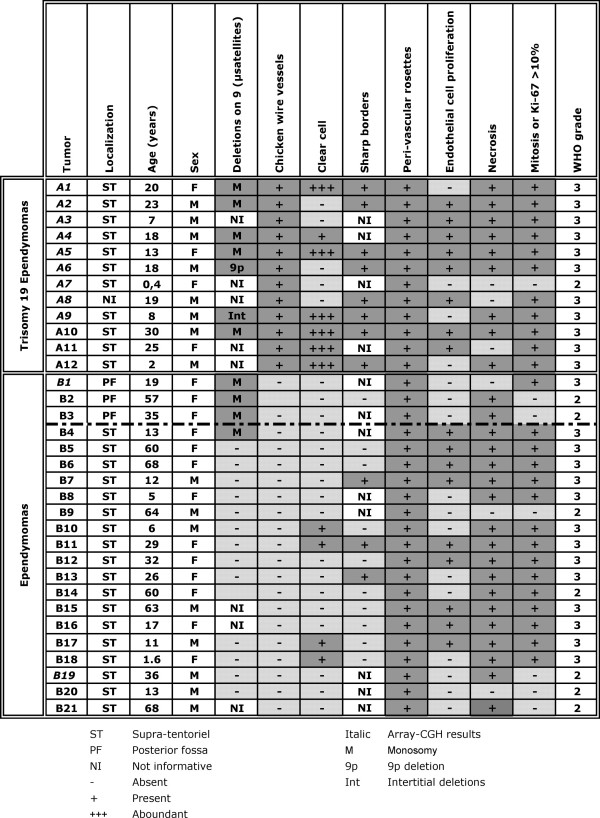
Clinico-pathological analysis of paraffin embedded supra-tentorial ependymal tumors, sub-ependymomas excluded, and of posterior fossa ependymomas presenting deletions of chromosome 9. Tumors are divided in between trisomy 19 ependymomas and ependymomas.

PCR amplicons with interpretable results for chromosome 9 markers used were obtained for 131 of the 149 tumors (89%). Fifty-nine were located in the posterior fossa, 27 in the supratentorial compartment, 17 in the spinal cord, 26 in the conus-cauda-filum, and 2 in an unknown localization. For 9 tumors, most of the 29 tested microsatellites on chromosome 9 were deleted (median 89%, with a range between 78% and 100%, Figure 1: A1-2, A4-5 and A10 and, B1-4). For an additional tumor, deletion was restricted to the p arm (Figure 1: A6) and in a last one, interstitial deletions of both arms were observed (Figure 1: A9). These 11 tumors were classified as having a deletion on chromosome 9 (8,5%). Three of them were located in the posterior fossa (Figure 1: B1-3), and presented classical ependymoma histology. The other 8 were located in the supratentorial compartment (Figure 1: A1-2, A4-6, A9-10 and B4). Seven of them demonstrated a compact architecture, regularly dispersed tumoral cells, chicken-wire vessels (Figure 1: A1-2, A4-6 and A9-10), and sometimes foci of clear cells (Figure 1: A1, A4-5, and A9-10). This prompted us to perform genomic profiling for tumors of such clinico-pathologic characteristics.

### Whole genome array-CGH

**Figure 2:**
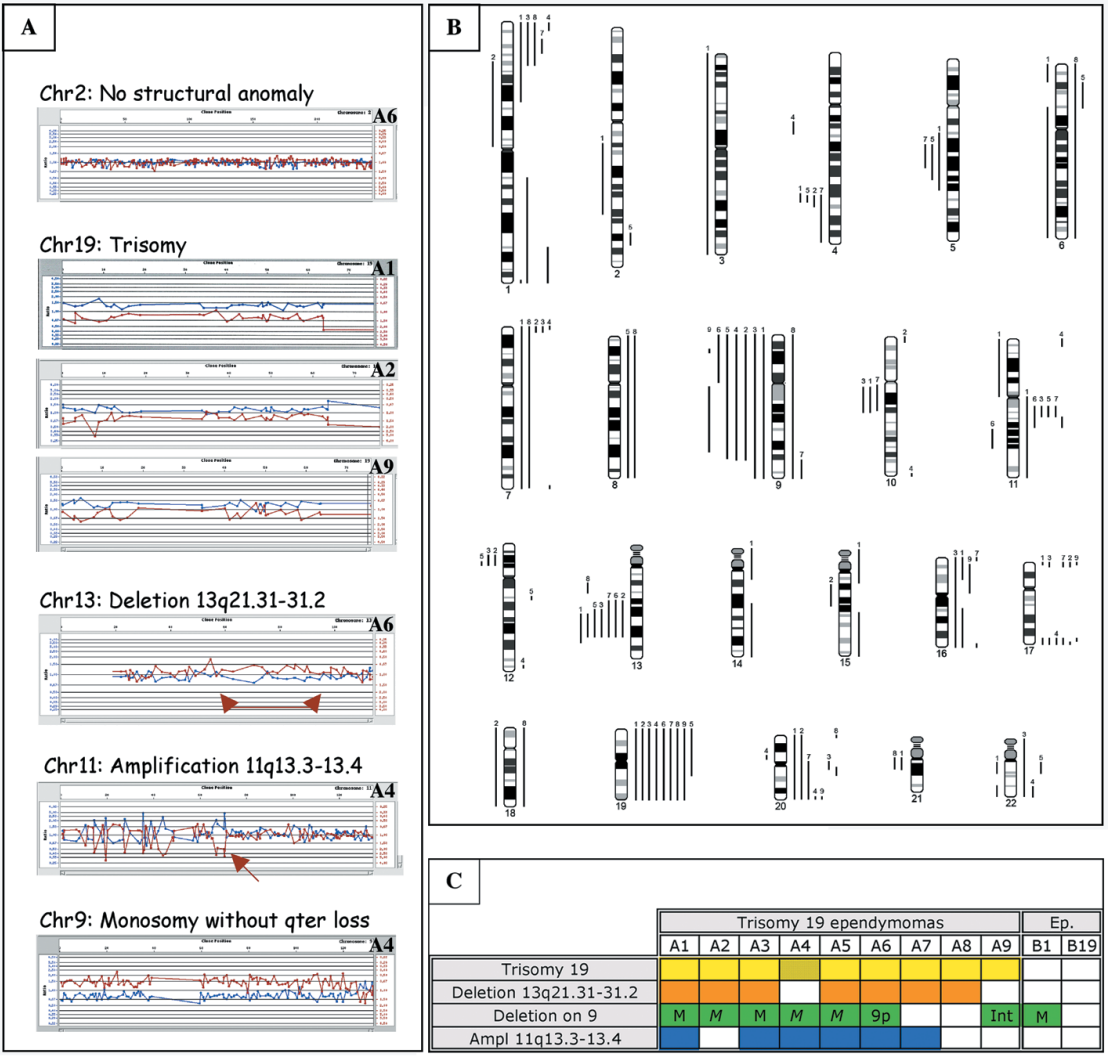
A) Array-CGH ideograms of the most frequently observed chromosomal anomalies in trisomy 19 ependymomas. Blue dots: ratio between tumor DNA and control DNA; red dots: ratio between control DNA and tumor DNA. Ratio of 1 indicates normal DNA content (presence of two alleles, chromosome 2: A6). Separation of the lines corresponds to gain of tumoral DNA (blue dots going up, chromosome 19: A1, A2 and A9 and, chromosome 11: A4) or loss of tumoral DNA (blue dots going down, chromosome 13: A6 and, chromosome 9: A4). B) Scheme of array-CGH results of trisomy 19 ependymomas. Altogether 118 genetic anomalies detected, mean: 13 per tumor, consisting of 74 gains (64%) and 44 losses (36%). Numbers correspond to tumors. C) Genetic alterations presented at least in 66% (6/9) of trisomy 19 ependymomas (A1-A9) compared to two controls (Ep.: B1 and B19). Trisomy 19 observed in all cases, although in one tumor (A4) the telomeric long arm was not amplified. Deletion of 13q21.31-31.2 and deletions on chromosme 9 (M: monosomy; M: monosomy without 9qter loss; 9p: 9p deletion and Int: intertitial p and q deletions) were found in 7/9 tumors (78%). Amplification of 11q13.3-13.4 was detected in 6/9 (66%) of the tumors.

Two of us (MMR and CG) re-reviewed the histology of the supra-tentorial ependymomas of the formalin-fixed and paraffin-embedded series to identify additional tumors with histology similar to that of the above described 7 tumors, and without chromosome 9 deletion. Five such tumors were identified bringing the total to twelve ependymomas with the histology of interest (marked A1-12, Figure 1). As DNA extracted from archival tumors is suitable for array-CGH [8-11], this technique was applied to 11 of these tumors for which enough DNA was available (Figure 1: A1-9 and A11-12). In addition, 3 ependymomas with classical histology (Figure 1: B1, B3 and B19) were profiled. Two of them presented a monosomy 9 by microsatellite analysis (Figure 1: B1 and B3). Results were obtained for 79% (11/14) of tumors, 9 with the histology of interest and 2 controls (Figure 2 and Figure 1: A1-9, B1 and B19). Genetic profiles divided them into two distinct groups.

The first one comprised of 9 tumors: 3 were recurrent and 6 primary (Figure 2C: A1-9 and Figure 1: A1-9). They all shared trisomy of chromosome 19 (Figure 2A, B, C), which was non-complete in some tumors (tumor A4, lack of telomeric q region amplification: Figure 2B, C). In this group, altogether 118 genetic anomalies were detected with a mean of 13 per tumor. They consisted of 74 gains (64%) and 44 losses (36%). Deletions on chromosome 9 were observed in 7/9 tumors (Figure 2A, B, C and Figure 1), limited to 9p in one tumor (tumor A6: Figure 2B, C and Figure 1), to interstitial deletions in another (tumor A9: Figure 2B, C and Figure 1) and, without 9qter loss in 3 (Figure 2B: A2, A4 and A5). An equally frequent deletion of 30 Mb was located at 13q21.31-13q31.2 (Figure 2A, B, C). Gain of a small region located at 11q13.3-13.4 was observed in 6 tumors (Figure 2A, B, C). Chromosome 17 telomeres were co-amplified in 5/9 tumors, and a sixth one had an amplification restricted to 17q telomere (Figure 2B). Chromosomes 1, 7, and 20 also frequently showed telomeric p or q amplifications (Figure 2B). Other whole chromosomal abnormalities, though less frequent, included monosomy 3 (1/9), trisomy 6 (1/9), trisomy 7 (2/9), trisomy 8 (2/9), monosomy 18 (1/9), trisomy 18 (1/9), trisomy 20 (1/9), and trisomy 22 (2/9) (Figure 2B).

The second group comprised of two tumors (Figure 2C: B1 and B19) neither one of which presented the recurrent chromosomal anomalies of the first group, with the exception of monosomy 9 in one tumor (B1). Nine genetic anomalies were observed per tumor consisting of 7 gains (39%) and 11 losses (61%).

### Whole genome profiling of frozen ependymal tumors by SNP-arrays

On the series of 24 frozen ependymomas, all presenting classical histological features of ependymoma, we observed only one ependymoma with partial trisomy of chromosome 19. This lesion was located at the spinal cord. It appeared as a compact tumor of low cellularity, devoid of mitosis, vascular proliferation and necrosis. This tumor, like the 23 other frozen ependymomas, did not fulfil clinico-histological aspect of the 9 ependymomas with a trisomy 19. Thus, altogether, 26 controls (24 frozen ependymomas + 2 paraffin-embedded ones, B1 and B19) were analyzed, and only one showed a partial trisomy 19 giving a p value < 0,001 (Fisher's exact test) for the presence of trisomy 19 in our first group of tumors.

### Characterization of trisomy 19 ependymoma

**Figure 3:**
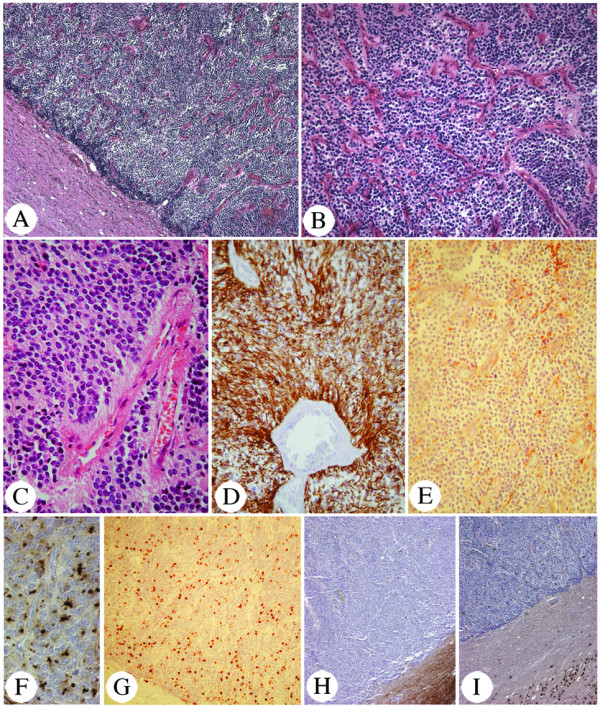
Histological characteristics of trisomy 19 ependymoma: compactness with clear cut border (A, HE, 30×); rich branched capillary network (B, HE, 90×); perivascular pseudorosettes, always observed, but can be rare (C, HE, 250×); GFAP positivity, always observed (D, GFAP, 125×) but can be focal with large GFAP negative area (E, GFAP, 125×); focal region of tumoral cells with intra-cytoplasmic EMA dots, always found (F, EMA, 310×); Ki67 >10% (G, Ki67, 60×); neuronal markers: negative in the tumor, positive in the surrounding brain parenchyma (H: NF, 30× and I: Neu-N, 30×).

**Figure 4:**
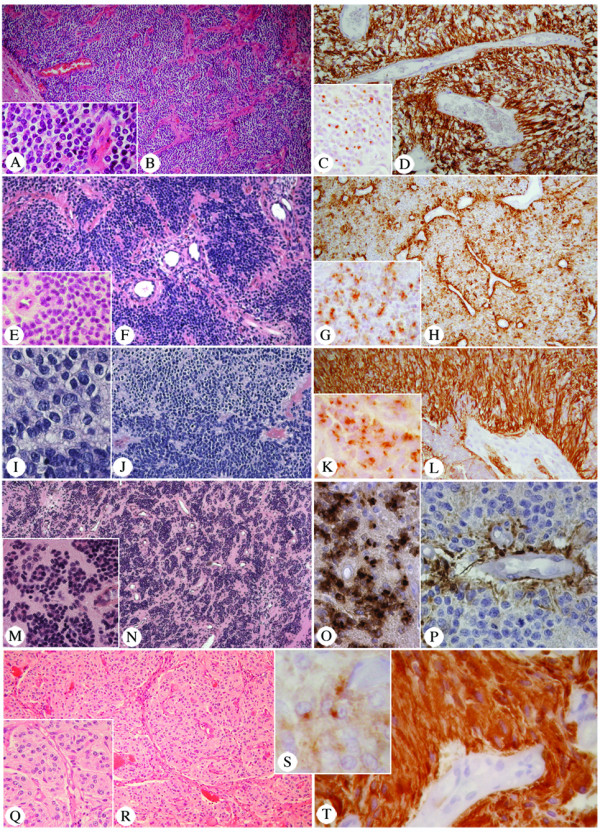
Three phenotypic variants of trisomy 19 ependymomas. Clear cells, indicative of clear cell ependymoma, a subgroup of trisomy 19 ependymoma (HE: A, 250× and B, 60×); PNET-like appearance, because tumoral cells almost devoid of cytoplasm, presence of rounded nuclei and nuclei-free regions not always centered on vessels (HE: E, 190×; F, 60×; I, 375×; J, 90×; M, 130× and N, 30×); and oligo-astrocytoma appearance, due to large eosinophilic tumoral cells and presence of chicken-wire vessels (HE: Q, 125× and R, 60×). Ependymal nature confirmed by GFAP positivity of end-feets (D, 125×; H, 60×; L, 160×; P, 375× and T, 375×) and intra-cytoplasmic EMA dots (C, 160×; G,160×; K, 250×, O, 250× and S, 500×). Pictures A, B, C and D correspond to tumor A1; pictures E, F, G and H to tumor A2; pictures I, J, K and L to tumor A4; pictures M, N, O and P to tumor A3, pictures Q, R, S and T to tumor A8.

The tumor group defined by the presence of trisomy 19 on array-CGH (A1-9), shared many clinico-pathological features (Figure 1: A1-9). In 8/9 cases, localization was supratentorial, for one it was unknown. The age at diagnosis ranged between 0.4 and 30 years, with a mean at 14 years and a median at 18 years (Figure 1). Histologically, they appeared compact with a well-demarcated brain-to-tumor border (Figure 3A). They presented a rich network of branched capillaries, reminiscent of the one of 1p/19q-deleted oligodendroglioma (chicken-wire vessels, Figure 3B, 4B, 4F and 4R), and tumoral cells were regularly distributed. In addition, pseudorosettes were always detected, meanwhile sometimes rare (Figure 3C). Microcalcifications were frequently encountered, and regions of classical ependymoma histology could be observed.

Tumoral cell phenotype varied between tumors and sometimes within the same tumor (Figure 4). In 3/9 tumors, most of the cells were ovoid with a clear perinuclear halo (Figure 1: A1, A5 and A9, and Figure 4A and 4B). In four tumors, the cells were almost devoid of cytoplasm with round nuclei (Figure 1: A2-4 and A6, and, Figure 4E, 4F, 4I, 4J, 4M and 4N). In these tumors, anucleated areas could be seen. They were not always centred on a vessel, imparting an initial impression of a neuroid-derived tumor. In one of these tumors, transition between neuroid to clear cell pattern was observed (Figure 1: A4 and Figure 4I and 4J). Finally, the remaining two tumors were composed of intermediate-to-large, ovoid or fusiform cells, with abundant cytoplasm that appeared palely stained or eosinophilic, suggestive of an oligo-astrocytic tumor (Figure 1: A7-8 and Figure 4Q and 4R).

The immuno-histochemical profile was similar in all trisomy 19 ependymomas. GFAP was always detected, although with intra and inter-tumoral variations. Some tumoral areas could be almost completely negative (Figure 3E), while others showed intensely labeled ependymal cells (Figure 3D, 4D, 4H, 4P and 4T). At least, perivascular positivity of cell end-feet was always found. EMA immunopositivity, appearing as intracytoplasmic dots, was observed focally in all tumors (Figure 3F, 4C, 4G, 4K, 4O and 4S). Immunolabeling for neurofilaments (Figure 3H) and NeuN (Figure 3I) was consistently negative in the tumor, although it was positive in the surrounding normal brain.

All but one ependymal tumor with trisomy 19 presented at least two of the following signs of anaplasia: (1) endothelial cell proliferation, (2) Ki-67 labeling index higher than 10% and (3) frequent mitotic figures (Figure 1). Therefore, they were considered as WHO grade III tumors.

## Discussion

We recognized a new genetico-histological association within ependymal tumors, the *trisomy 19 ependymoma*. Most of these tumors are supra-tentorial WHO grade III tumors of the young. Trisomy of chromosome 19 is frequently associated with deletion of 13q21.31-31.2, three copies of 11q13.3-13.4, and/or deletions on chromosome 9. The histological hallmark is a prominent branched capillary network around which tumoral cells are regularly dispersed. Clear cells may be present in these compact lesions, evoking the diagnosis of clear cell ependymoma. Trisomy 19 ependymomas have an immunohistological profile of ependymal tumors: positive for glial fibrillary acidic protein (GFAP) and epithelial membrane antigen (EMA), and negative for neuronal markers.

In the "cancer chromosomes database" [12], we calculated chromosomal trisomy to occur with a mean frequency of 4% among all tumors (n = 50,380), which was comparable for chromosome 19 (4.2%). In brain tumors (n = 1644), trisomy 19 occurred in 6%, but only in about 3% of meningiomas (n = 817) and, interestingly in about 9% of astrocytomas (n = 569) and ependymomas (n = 111). This more frequent observation in glial tumors suggests an ethiopathogenic role within this group of tumors. Our results linked trisomy 19 to a subset of supra-tentorial ependymomas that we could recognise on histological criteria. Furthermore, in the two control series, we observed only one tumor with a partial chromosome 19 trisomy (3,8%), giving a p value < 0,001 for the presence of trisomy 19 in our tumor group of interest. Because trisomy 19 appears non-complete in some of our ependymomas, it has to be looked for using a technique which profiles the entire chromosome 19 for copy number alterations.

The other copy changes are helpful in recognition of trisomy 19 ependymomas. Interstitial deletion of 13q and deletions on chromosome 9 were identified in 7/9 (78%) of the tumors. They were observed associated with trisomy 19 only once and twice respectively in the NCBI database (frequency of 0,9% and 1,8%) [12], and not in our control. Similarly, association between trisomy 19 and amplification of 11q13.3-13.4, which was observed in 6/9 (66%) of our trisomy 19 ependymomas, was rarely reported in the NCBI database (4,5%) and not found in the control series. All of our trisomy 19 ependymomas presented at least one of these associated anomalies (100%) and 7/9 (78%) two of them.

The WHO classification describes clear cell ependymomas as having an "oligodendroglia-like appearance with clear perinuclear haloes" [1]. This definition highlights the importance of two histological features: clear cells and chicken-wire vessels. The combination of our genetic and histological data emphasized the latter. Chicken-wire vessels were constantly observed in ependymal tumors bearing trisomy 19, whereas clear cells were not. Thus clear cell ependymoma are a subgroup of trisomy 19 ependymoma.

Previously, trisomy 19 ependymomas may have been reported as haemangioblastoma, PNET, central neurocytoma, oligodendroglioma or oligo-astrocytoma [13-16]. Amongst them, haemangioblastoma is the only one with a reticulin rich stroma. Neuronal markers are positive in PNET and central neurocytoma, but negative in trisomy 19 ependymoma. GFAP and EMA, which are negative in neuronal tumors, are positive in ependymal tumors, although this can be focal [14,17]. Because of this, differential diagnosis between trisomy 19 ependymomas and oligodendroglioma/oligoastrocytoma may be difficult. Genetic analysis can help. Deletion of 1p and 19q is restricted to oligodendroglioma and oligo-astrocytoma [18,19], whereas trisomy 19 suggests trisomy 19 ependymoma.

Rickert and co-workers recently analyzed by classical CGH a series of 13 clear cell ependymomas [20]. They pinpointed monosomy 9 to be always associated with WHO grade III and sometimes with WHO grade II clear cell ependymoma. They defined clear cell ependymomas as comprising of at least 50% of clear cells. In our series of trisomy 19 ependymomas, 3/9 tumors contained similar percentage of clear cells (Figure 1: A1, A5 and A9), one less (Figure 1: A4), and five none (Figure 1: A2-3, A6-8). All 4 trisomy 19 ependymomas with clear cells had an additional chromosome 9 deletion, in agreement with the published series. Amongst the 5 trisomy 19 ependymomas devoid of clear cells, three had a deletion on chromosome 9. Moreover, chromosome 9 deletion was also observed in other subtypes of ependymomas (Figure 1: B1-4). There is a technical difference as well. The total number of reported chromosomal anomalies was 2,7 per tumor in the Rickert analysis, whereas we demonstrated a mean of 13 anomalies per tumor, illustrating the higher resolution of array-CGH. In addition, Rickert's series did not demonstrate anomalies of chromosome 19, whereas trisomies of this chromosome were observed in a series of clear cell ependymomas analyzed by FISH [21]. Presence of high amount of heterochromatin in chromosome 19 is known to induce false positive and negative CGH results, a reason why this chromosome is often difficult to interpret and frequently excluded from classical CGH analysis [22,23].

**Figure 5:**
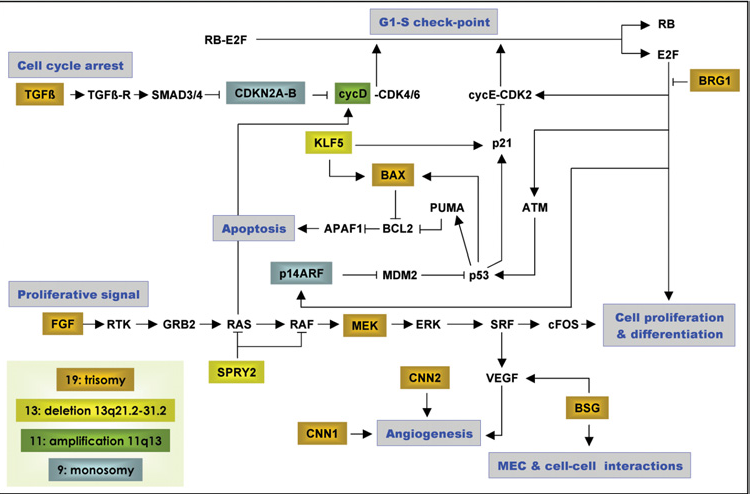
Scheme of pathways involved in trisomy 19 ependymoma. Cancer occurs because of accumulation of defects implying cell cycle, cell proliferation, differentiation, apoptosis, cell interaction with surrounding stroma and angiogenesis. All these pathways could be deregulated when considering genes located in the 4 chromosomic regions most frequently altered in trisomy 19 ependymomas.

Cancer arises from accumulations of genetic changes in pathways involved in cell cycle, cell proliferation, apoptosis, angiogenesis and interaction with extracellular matrix [24]. Interestingly, all these biological processes can already be altered by genes located on chromosome 19 (Figure 5) [25-41]. The 3 other chromosomic regions involved in trisomy 19 ependymomas could reinforce potential deregulation of these pathways (Figure 5) [35-44]. Furthermore, epigenetic changes are of importance. Chromosome 19 contains *BRG1 *(19p13.2), and chromosome 9 *BRM *(9p24.3). These genes are ATPase subunits of *SWI/SNF *complex, one of the two enzymes implicated in chromatine remodeling and promotor methylation [45].

## Conclusion

We describe a new subgroup of ependymal tumors, the *trisomy 19 ependymoma*, which bears specific clinico-histological characteristics. This newly identified association is a significant additional argument for considering disparity in tumorigenesis pathways involved in ependymal tumors as suggested by Lukashova and co-authors [46]. Differences have also been illustrated on differential expression of DAL-1 and NF2 between intracranial and spinal cord ependymomas [47], methylation of RASSF1A and TRAIL pathway-related genes in childhood intracranial ependymomas [48], and methylation of 9p21 tumor suppressor genes following clinico-histological parameters of ependymal tumors [7]. Further genetico-histological dissection of tumors is needed for development of targeted oncological practice.

## Methods

### Tumor samples and DNA extraction

Two independent series of ependymal tumors were analyzed. None of the tumors were simultaneously included in both series. The first one comprised of 24 frozen ependymal tumors and the second of 149 formalin-fixed and paraffin-embedded ones. Both series were retrieved from the archives of 9 neuropathological centers based on their original diagnosis of ependymomas (Institute of Neurology, London, UK; Hospital Roger Salingro, Lille, France; Laboratory of neuropathology, CHU-Caen, France; St. Luc hospital, Université catholique de Louvain, Belgium; La Timone's Hospital, AP-HM tumor bank, Marseille, France; Hospital Erasme, Université libre de Bruxelles, Belgium; CHU Kremlin-Bicêtre, Paris, France; CHU-Lariboisière, Paris, France; University of Newcastle, Newcastle, UK). Before inclusion in the study, all tumor diagnoses were revised following the latest WHO classification [1]. The study was approved by the ethics committee of the Medical Faculty of Université catholique de Louvain, Brussels, Belgium.

The 24 frozen ependymomas were obtained from 23 patients, 18 corresponding to primary tumors and 6 to recurrent tumors. For one patient, we received both the primary and the recurrent tumor. Age at operation varied between 4 months and 63 years with a mean age of 27.5 years and a mediane age of 33 years. Histologically, the tumors were classified as myxopapillary ependymomas (WHO grade I, n = 2, 8%), ependymomas (WHO grade II, n = 11, 46%) and anaplastic ependymomas (WHO grade III, n = 11, 46%). Thirteen were located in the spinal cord (54%), 4 in the posterior fossa (17%) and 7 in the supratentorial compartment (29%). This series was used for single nucleotide polymorphism (SNP) microarrays analyses.

The 149 formalin-fixed and paraffin-embedded tumors were obtained from 146 patients. In three patients, tumors were collected from 2 consecutive surgical resections. In 17 patients samples were available only from the recurrence. Age at operation varied from 3 months to 80.6 years, with a mean of 29.4 years and a median of 27.4 years. More precisely, 19 patients were aged between 0 and 3 years (13%), 29 between 3 and 15 years (19%), and 100 were older than 15 years (67%). For 1 patient, the age was unknown (<1%). Histologically, the tumors were classified as subependymomas (WHO grade I, n = 15, 10%), myxopapillary ependymomas (WHO grade I, n = 28, 19%), ependymomas (WHO grade II, n = 63, 42%), and anaplastic ependymomas (WHO grade III, n = 43, 29%). Forty-nine were located in the spinal cord (32.9%), 63 in the posterior fossa (42.3%), 33 in the supra-tentorial compartment (22.1%), and 4 in an unknown location (2.7%) This series was used for microsatellite analysis and array-CGH.

For microsatellite analysis, DNA was extracted from formalin-fixed and paraffin-embedded tumors using QIAamp DNA Mini Kit (Qiagen, Westburg, Leusden, Holland) after deparaffinization of tumor shaves with xylene. This was performed following a tissue dissection step if section contained more than 10% of normal brain parenchyma. For 6 patients, enough normal tissue was obtained to be used as control DNA.

For array-CGH, tumoral DNA was extracted from formalin fixed and paraffin embedded tumors using PUREGENE DNA Purification Kit (Gentra Systems, Minneapolis, Minnesota, USA) after deparaffinization of tumoral shaves using xylene. Control DNAs were extracted from blood samples of healthy individuals. Pools of either 5 males or 5 females were constituted. Absence of DNA anomaly in both pools was confirmed by hybridization of male pool against female pool. For SNP chip analysis, DNA was extracted from frozen tumors as described for array-CGH, but without deparaffinization.

### Immunohistochemistry

Immunohistochemistry was performed following classical protocols. Briefly, 5 μm sections were deparaffinized using Histosafe (Yvsolab, Beerse, Belgium), rehydrated in propanol and finally water. After blocking endogenous peroxydase by incubating sections for 30 min in 0.3% H_2_O_2_, antigens were retrieved using a citrate buffer pH 5.7 at 95°C for 95 min. Non-specific binding was inhibited under 10% normal goat serum and 1% bovine serum albumin. The slides were subsequently incubated overnight at 4°C with the following primary antibodies: Glial Fibrillary Acidic Protein (GFAP, rabbit polyclonal obtained from Dako, dilution 1/2000), Epithelial Membrane Antigen (EMA, mouse monoclonal, Neomarkers, dilution 1/200), Neurofilaments 68 and 200 (NF, mouse monoclonal, Sigma and Boehringer, used mixed together at 1/200 and 1/25 dilution), Neuron-specific Nuclear Protein (NeuN, mouse monoclonal, Chemicon, dilution 1/100), and Ki-67 (mouse monoclonal, Dako, dilution 1/100). After a wash with Tris-HCl 0.05 M, pH 7.4, either a biotinylated anti-mouse (Vector, dilution 1/500) or anti-rabbit (Boerhinger, dilution 1/500) secondary antibody was applied on the sections for 30 min at room temperature. The slides were then washed with Tris-buffer and incubated with a streptavidin-peroxydase complex (Roche, dilution 1/100) for 30 min at room temperature. After a wash, the chromogen was revealed using 0.05% DAB (Fluka) in PBS buffer (pH 7.2) with 0,01% H_2_O_2 _during 10 min. The slides were rinsed with water, counterstained with Mayer's Hematoxylin and mounted.

### Microsatellite analysis

Twenty-nine microsatellites of chromosome 9 were chosen from the Human MapPairsTM Genome-Wide Screening Set 8 (Weber set, Research Genetics) or 9 (Li-Cor, Westburg, the Netherlands), or in the Unified Database for Human Genome Mapping on the basis of their map position [49]. The latter were synthesized by Eurogentec (Belgium) or MWG (Germany). Of the 29 microsatellites, 23 were located on 9p (D9S917, D9S288, D9S1810, D9S2169, D9S2156, located at 9p24; D9S775, D9S921, D9S168, D9S269, D9S254, located at 9p23; D9S285, D9S156, D9S157, D9S925, located at 9p22; D9S162, D9S1749, D9S1748, D9S171, D9S1679, D9S1121, D9S251, D9S1118, and D9S1788, located at 9p21) and 6 were located on 9q (D9S301, D9S1122, D9S922, located at 9q21; D9S930, located at 9q32; D9S934, and D9S1825, located at 9q33).

For amplification of microsatellites with primers of the Weber set 8, one of the two primers was end-labeled with gamma-32P ATP (Amersham-Pharmacia), using T4 polynucleotide kinase (TAKARA). PCRs were performed in a final volume of 10 μl containing 18 ng of template DNA, 0.6 μM of each primer, 1× Biotools buffer, 0.2 mM of each dNTP, and 0.025 U/μL Biotools DNA polymerase (Lab Systems, Belgium). After 5-minutes denaturation at 95°C, 35 cycles were realized with 94°C for 40 seconds, 55°C for 50 seconds and 72°C for 50 seconds, with a final extension of 5 minutes at 72°C. For amplification of the Weber set 9 (Westburg, Holland) and other fluorescent markers modified with IRD 700 or IRD 800 fluorochromes (MWG, Germany), PCRs were carried out as above, except that 36 ng of template DNA and 2 to 5 mM MgCl2 were used. All PCRs were performed in duplicate.

To visualize alleles of microsatellites, amplicons were heat denatured (95°C for 5 minutes) after addition of 10 μl of a denaturing loading buffer. 2.5 μl of the obtained solution was run on gel. For radio-active markers, a 5% polyacrylamide denaturing gel was used. The gels were subsequently vacuum-dried and exposed overnight on Kodak X-OMAT AR films (Amersham-Pharmacia, Belgium). For fluorescent markers, a 6,5% SequaGel XR gel (National Diagnostics) was used on a Li-Cor Gene Readir 4200 DNA Analyzer (Westburg, the Netherlands), operated by E-Seq DNA Sequencing and Analysis software (version 1.0 Westburg, the Netherlands). The analysis was made with Gene ImagIR software (version 4.0, Westburg, the Netherlands).

Interpretation of microsatellite results was performed as previously described [50]. Briefly, the status of chromosome 9, for a given tumor, was only taken into account when more than 70% of the tested microsatellites gave interpretable amplicons. In addition, to be considered monosomic, 90% of the amplicons had to reveal either the presence of a single allele or an important difference in intensity between the two alleles.

### Array-CGH

Array-CGH was performed according to manufacturer's instructions (Spectral Genomics, USA) with few modifications. After DNA extraction and purification with the Qiagen PCR purification kit (Westburg, Holland), the DNAs were labeled with Cy3-dCTP and Cy5-dCTP (Amersham) using the Gibco/BRL Bioprime DNA labeling kit. Labeled test and reference DNA were subsequently mixed in the proportion of 1.2–1.4 μg of Cy3 labeled DNA to 1 μg of Cy5 labeled DNA. Spectral Hybridization buffer I containing cot-1 DNA was added to the mixture. DNAs were precipitated with 5 M NaCl/isopropanol solution, rinsed with 70% ethanol and air-dried. The precipitate was resuspended in water and mixed with the Spectral Hybridization buffer II. Denaturation at 72°C for 10 minutes was followed by incubation of 10 min on ice and subsequently of 30 min at 37°C. Hybridization on the array (HU 1K BAC Array, Spectral Genomics, USA) was performed in a hybridization chamber at 37°C for 16 hours. To increase specificity, each hybridization was also performed by flipping tumor and control fluorochromes. The arrays were rinsed in the 5 subsequent solutions: 2 × SSC, 0.5% SDS for 20 min at 45°C; 2 × SSC, 50% formamide for 20 min at 45°C; 2 × SSC, 0.1 × NP-40 for 20 min at 45°C, 0.2 × SSC for 10 min at 45°C, and a brief rinse in H_2_O. The arrays were scanned and analyzed using the Spectralware software (version 2,0).

### Single Nucleotide Polymorphism microarrays

The Affymetrix high-density (50 K) oligonucleotide array-based SNP genotyping was performed according to the standard protocol for Affymetrix GeneChip Mapping 100 K arrays (Affymetrix, Inc.). Only tumors with a call rate higher than 94% were included in the analysis. Briefly, 250 ng of genomic DNA was digested by a restriction enzyme (XbaI), which allowed to ligate an adaptor used for PCR primer annealing for the subsequent whole genome amplification. The obtained PCR products were size-restricted by digesting with DNAseI. This fragmented DNA was labeled with a biotinylated nucleotide analogue and hybridised to the microarray. Hybridised fragments of tumoral DNA were revealed using a three step detection system constituted of a first streptavidin-phycoerythrin binding, followed by an anti-streptavidin biotinylated antibody incubation and, lastly, a final streptavidin-phycoerythrin step. After scanning of the array, the SNPs were genotyped by GeneChip DNA Analysis Software (GDAS, version 3.0.2.8; Affymetrix, Inc.). Raw signals (genotype and intensity data of the SNP probes) were exported from the Affymetrix platform and analyzed for copy number alterations using the Copy Number Analyser for GeneChip (CNAG, version 1.0) [51] and dCHIP (version 2005) [52] softwares.

## Competing interests

The authors declare that they have no competing interests.

## Authors' contributions

ER performed the microsatellite analysis and participated to draft the manuscript; TP performed the SNP chip study and participated in drafting of the manuscript; CG, FS, MMR, DF and DE participated in the histological analysis and provided tumors; IS, CL, FC and JM provided tumors; CG performed the array-CGH study; MV and CG conceived the study, and participated in its design, analysis of data and coordination. All authors read and approved the final manuscript.
